# Blood Levels of Dendritic Cell Populations in Patients with Moderate to Severe Traumatic Brain Injury

**DOI:** 10.1177/2689288X251396704

**Published:** 2025-11-12

**Authors:** Patrik Fridh, Ebba Katsler, Niklas Marklund, Sara Mangsbo, Ted Ebendal

**Affiliations:** ^1^ Department of Pharmacy, Uppsala University, Uppsala, Sweden.; ^2^ Department of Clinical Sciences Lund, Neurosurgery, Lund University, and Skåne University Hospital, Lund, Sweden.

**Keywords:** blood inflammatory profile, classical dendritic cells type 2 (cDC2), inflammatory monocyte-derived dendritic cell (moDC), neuroinflammation, TBI

## Abstract

Traumatic brain injury (TBI) leads to intracerebral inflammation involving resident microglial cells and astrocytes as well as invading peripheral dendritic cells (DCs), monocytes, and neutrophils. However, the profile of immune blood cells activated by TBI remains poorly defined. Several animal models showing invasion of circulating classical dendritic cells type 2 (cDC2s) to the traumatically injured brain have been found, resulting in exacerbated neurological outcomes. In TBI patients, increased levels of chemokine CCL2, attracting cDC2 cells, have been linked to poor recovery. In the present study, blood samples from healthy blood donors (*n* = 11) were compared with blood from TBI patients (*n* = 15) at day 1 and day 3 after admission for neurointensive care stored in two tested freezing media (eight patients using Cytodelics, seven patients using CryoStor CS10) for analyses by flow cytometry. Reference blood was collected from random healthy blood donors (7 with Cytodelics, 4 CryoStor CS10). Flow cytometry excluded T-cells, B-cells, and natural killer cells by a panel of CD3, CD19, CD20, and CD56 antibodies. To identify DCs and inflammatory monocytes, antibodies to CD11c, CD1c, CD141, HLA-DR, and CD14 labeled with specific fluorochromes were added to the thawed blood samples. Neutrophils were analyzed by separate runs of flow cytometry using a CD66b antibody. Despite some differences depending on the freezing medium used, the percentage of classical DCs type 2 (cDC2; CD14-, CD11c high, CD1c+) remained unchanged from healthy controls at day 1 after admission but increased significantly (*p* = 0.014) from day 1 until day 3 after TBI. In contrast, levels of classical DCs type 1, inflammatory monocyte-derived DCs, or neutrophils were not altered. Thus, our preliminary data, in addition to previous animal model data, suggest a role for circulating cDC2 cells contributing negatively to the pathophysiology of TBI.

## Introduction

Traumatic brain injury (TBI) results from an external force disrupting normal brain functions. It is a leading cause of death and disability, estimated to occur in well over 50 million patients globally each year, and leads to two percent of the world’s population living with neurological impairments.^1–3^ The initial trauma results in injury to neuronal and glial cells and leads to hemorrhage due to vascular injuries. The primary injury is exacerbated by a complex of prolonged secondary molecular and cellular mechanisms.^4–7^ An important, yet complex, secondary injury mechanism is the activation of inflammatory responses that includes the release of cytokines, chemokines, and damage-associated molecular patterns. TBI activates resident endogenous microglia and astrocytes but, in addition, attracts inflammatory cells from the blood circulation to enter the brain both in humans^8^ and rodents.^9–11^ In particular, the level of the chemokine C-C motif ligand 2 (CCL2, also known as monocyte chemoattractant protein 1, MCP1) is rapidly upregulated in cortical areas following TBI and stimulates invasion of immune cells expressing the G-protein-coupled cognate C-C chemokine type 2 receptor (CCR2).^6–9,12^

The present study examined the possible early increase of blood-borne inflammatory cells such as dendritic cells (DCs),^13^ monocytes, and neutrophils in response to chemoattractant signals in patients admitted for neurointensive care with traumatic brain injuries. In order to collect clinical blood samples for later analysis by flow cytometry taken from patients and healthy volunteers, we applied of two regimens of blood cell preservation, either Cytodelics preservation of cells or freezing with the reagent CryoStor CS10, to evaluate that we did not impact innate cell numbers by preservation method only. The study was undertaken to gain knowledge on changes over time in the percentage of specific cell populations in whole blood of TBI patients.

## Methods

### Blood samples

Whole blood samples were collected from totally 18 patients with moderate to severe TBI admitted to the Neurosurgical Department, Skåne University Hospital, Lund, Sweden (Table 1), at day 1 and day 3 after admittance when possible. Patients included had a Glasgow Coma Scale (GCS) score of 3–12.^14^ Neuroimaging characteristics of the injury gathered from computed tomography (CT) scans were based on the Marshall classification.^15–16^ The worst CT-scan obtained during the stay was analyzed, i.e., the CT scan showing the most lesions and/or midline shift at the level of the thalami, effacement of the basal cisterns and injury type was also determined.^17^

**Table 1.** TBI Patients and Healthy Blood Donors Recruited to the Study

Freezing	Donor	Patient number	Sex (F/M)	Age (years)	GCS	Marshall classification	CNS injuries	Midline shift	Day 1	Day 2	Day 3	GOSE
Medium								> 5 mm				
Cytodelics	Patient	1	M	69	3–8	III/VI	Skull fracture, contusions, tSAH, tICH	No	X		X	1
	Patient	3	M	77	15	II/II	contusion, tSAH	No	X		X	8
	Patient	5	M	64	15	IV/IV	Skull fracture, aSDH^f^	Yes	X		X	8
	Patient	6	M	70	10	II/II	Skull fracture, contusions, tSAH, tICH, aSDH, fracture at Th11, L3, L4, medullary transection	No	X		X	4
	Patient	7	M	65	10	III/V	tICH, aSDH, hygroma	Yes	X		X	1
	Patient	8	M	45	11c	II/II	Skull fracture, contusions, tSAH, EDH	No	X		X	6
	Patient	9	F	20	NR^h^	II/II	Skull fracture, contusions, tSAH, SDH, EDH	No	X		X	NR
	Patient	10	F	64	9	II/VI	Skull fracture, contusion, tSAH, tICH, aSDH	Yes	X		X	1
	Healthy Donors	*n* = 7	NR	NR								
CryoStor CS10	Patient	12	M	39	13	II/V	Skull fracture, contusion, EDH.	Yes	X		X	5
	Patient	14	M	68	15	II/V	Skull fracture, IVH^i^, tSAH, lacerations	No	X		X	6
	Patient	15	F	74	12	V/V	aSDH	Yes	X		X	4
	Patient	16	M	65	13	VI/VI	Skull fracture, contusions, tSAH, tICH	Yes	X		X	4
	Patient	17	M	25	3	V/V	Contusions, aSDH,	Yes	X			1
	Patient	18	F	71	15	II/II	tSAH	No	X			8
	Patient	19	M	68	15	II/II	tSAH	No		X		8
	Healthy Donors	*n* = 4	NR	NR								

GCS, Glasgow Coma Scale, Marshall classification, at arrival and highest during hospitalization, CNS, central nervous system; tSAH, traumatic subdural hematoma; tICH, traumatic intracerebral hemorrhage; aSDH, acute subdural hematoma; EDH, epidural hematoma; NR, not reported, IVH, intraventricular hemorrhage; F, female patient; M, male patient.

Samples from a first cohort of patients were frozen in the Cytodelics reagent. Blood from a second group of TBI patients, later arrivals to the neurointensive care unit, was frozen in the alternative preservation medium CryoStor CS10. Patients originally numbered 11 and 12 were excluded since their injuries strictly affected the spinal cord. Patient 2 was omitted from the final analysis since the blood samples were consumed in the process of optimizing our flow cytometry strategy; thus, the total number of TBI patients included in our study is 15 (Table 1). Permission to study blood samples was approved by the Regional Ethics Committee in Lund to Niklas Marklund (Dnr 2017/469, Dnr 2017/1049, and Dnr 2022-07096-02, Lund University, Regional Ethics Board Lund), and next of kin signed the consent since the patients were not capable of consenting. As a reference, we purchased and analyzed heparinized whole blood samples from healthy blood donors (*n* = 11) from Uppsala University Hospital.

### Cytodelics

Cryovials (Thermo Fisher Scientific) were prefilled with 600 μL of Cytodelics® stabilizer (Cytodelics AB, Södertälje, Sweden) before 600 μL of whole blood was added. The cryovials were frozen according to the manufacturer’s protocol and stored at −80°C. For flow cytometry analyses, frozen samples were thawed in a water bath at 37°C by swirling the cryovials for 1–2 min or until approximately 90% of the ice melted. A 50 mL Falcon tube (Sarstedt Inc.) received fixation buffer by combining 3 mL of Cytodelics Fix-Concentrate and 3 mL of Cytodelics Fix-Diluent. The thawed blood was added to the tubes, incubated at room temperature (RT) for 15 min, and vortexed multiple times during the incubation time, whereafter 24 mL of Cytodelics lysis buffer (diluted to 1x with Milli-Q H_2_O) was added for incubation in the dark for an additional 15 min before centrifugation for 5 min at RT and 400 × *g*. After carefully removing the supernatant, the pellet was first washed with 24 mL of 1x Cytodelics wash buffer and then with 5 mL of phosphate buffered saline (PBS) (Gibco) including 1% bovine serum albumin (BSA) (Sigma-Aldrich). The pellet was resuspended with 1.2 mL of PBS + 1% BSA, transferred onto a 96-well V-bottom plate (Sarstedt Inc.), and centrifuged. The supernatant was carefully removed before staining with fluorescent-labeled antibodies.

### CryoStor CS10

Cryovials prefilled with 1.2 mL of CryoStor®CS10 (Stemcell Technologies, Vancouver, British Colombia, Canada) received 600 μL of whole blood for freezing and storage at −80°C. The thawing process for flow analyses was by swirling the cryovials in a water bath at 37°C for 1–2 min. Before centrifugation for 5 min at RT and 300 × *g*, 18 mL of prewarmed PBS was added to a 50 mL Falcon tube before the thawed whole blood. After removing the supernatant, 12 mL of RBC lysis buffer (eBioscience) was added and incubated for 15 min at RT in the dark. After centrifugation for 5 min at RT and 400 × *g*, the supernatant was discarded and washed once with 5 mL of PBS. The pellet was resuspended in 1.2 mL of PBS + 1% BSA and added to a 96-well V-bottom plate. The plates were centrifuged, and supernatants were removed before staining with fluorescent antibodies.

### Flow cytometry

Thawed blood samples were analyzed as technical replicates and stained with antibodies from BioLegend (Table 2). Human TruStain FcX (BioLegend) was added to the wells and incubated for 10 min at 4°C in the dark. 50 uL of antibody cocktail (diluted in PBS + 1% BSA) was added to each well and incubated for 20 min at 4°C in the dark. Wells were topped up with PBS + 1% BSA, centrifuged for 5 min at RT and 400 × *g* before the supernatant was discarded. The pellet was resuspended in 100 uL of PBS + 1% BSA, and samples were analyzed by CytoFLEX® Flow Cytometer (Beckman Coulter Life Sciences.

**Table 2.** Antibodies Used for Flow Cytometry of Frozen/Thawed Human Blood Cells

Antibody	Host species, isotype	Fluorochrome	Clone	Dilution
Lineage panel				
Anti-human CD3	Mouse, IgG1	Pacific Blue	SK7	1:100
Anti-human CD19	Mouse, IgG1	Pacific Blue	HIB19	1:100
Anti-human human CD20	Mouse, IgG2b	Pacific Blue	2H7	1:100
Anti-human CD56	Mouse, IgG2a	Pacific Blue	MEM-188	1:100
Dendritic cells panel				
Anti-human HLA-DR	Mouse, IgG2a	PerCP/Cy5.5	L243	1:100
Anti-human CD14	Mouse, IgG2a	BV605	M5E2	1:100
Anti-human CD11c	Mouse, IgG1	PE	Bu15	1:100
Anti-human CD1c	Mouse, IgG1	PE/Cy7	L161	1:100
Anti-human CD141	Mouse, IgG1	BV785	M80	1:100
Neutrophil staining				
Anti-human CD66b	Mouse, IgM	APC/Cy7	G10F5	1:100

**FIG. 1.**
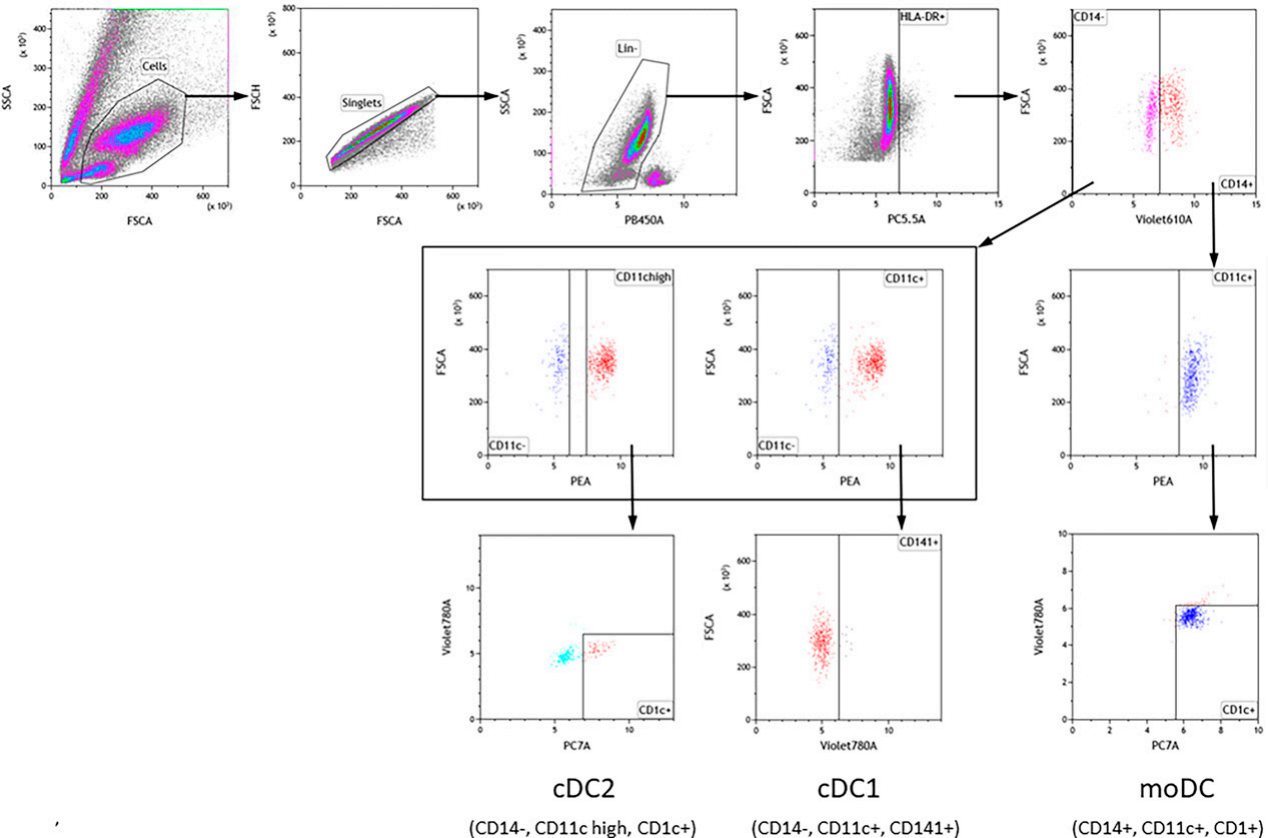
Flow cytometry. Gating strategies to identify classical dendritic cells type 2 (cDC2), classical dendritic cells type 1 (cDC1) and inflammatory monocyte-derived dendritic cells (moDC) in blood samples from TBI patients and healthy blood donors. The cDC2 cells (CD14-, CD11c high and CD1+) are seen in the bottom left quadrant, the cDC1 cells (CD14-, CD11c+, CD141+) bottom middle and moDC cells (CD14+, CD11c+, CD1+) appear at the extreme bottom left area.

Lineage staining was applied as a dump channel, where lineage-positive cell populations were eliminated to discard T-cells, B-cells, and natural killer (NK) cells discarded by the use of anti-CD3, anti-CD19, anti-CD20, and anti-CD56 antibodies, all labelled by Pacific Blue. The panel of specific cell surface markers (Table 2) included mouse monoclonal anti-human HLA-DR-PerCP/Cy5.5 (clone L243), anti-human CD14-Brilliant Violet 605 (clone M5E2), anti-human CD11c-Phycoerythrin (clone Bu15), anti-human CD1c-Phycoerythrin/Cy7 (clone L161), and mouse anti-human CD141-Brilliant Violet 785 (clone M80). As controls, fluorescent minus one (FMO), single stain (SS), and unstained samples (US) were used. Kaluza Analysis Software 2.1 (Beckman Coulter Life Sciences) was used for gating as shown in Figure 1.

All samples were analyzed, normally as quadruple technical replicates. As controls of master mix (MM) samples, FMO, SS, and US were used. Neutrophils were examined in separate runs of flow cytometry by the use of anti-human CD66b (Table 2). Flow data were imported and processed using the cyCombine R package (v.0.1.8), normalized and batch-corrected using self-organizing maps, followed by evaluation of batch correction performance using Earth Mover’s Distance and Median Absolute Deviation.

### Data and statistical analysis

All graphs and statistical analyses were made using Prism 10 (GraphPad Software Inc.). For determining the normality of the data sets, the Shapiro-Wilk test was used, while the variance was determined with an *F* test. Individual data points are shown as box plots. Statistical comparisons between days based on the mean were carried out with either Student’s *t*-test or the Mann–Whitney U test. For individual patients, pairwise comparisons between days were performed with the Wilcoxon test. The significance threshold was set at *p* ≤ 0.05 and *p*-values indicated in the Figures as non-significant (ns) if *p*-values >0.05, *p ≤ 0.05, or **p ≤ 0.01.

## Results

This study was undertaken to gain knowledge on changes within the first days of specific inflammatory cell populations in whole blood of TBI patients analyzed by flow cytometry. Blood samples were collected on day 1 and day 3 after hospital admission, with the exception of three patients of the 15 included (Table 1). As expected, a significant correlation was found between the GCS and GOSE scores (*p* < 0.001), whereas the blood levels of cDC2 cells in individual patients did not closely reflect these scores and did not reach statistical significance in view of the low number of included patients.

The samples were analyzed by flow cytometry using a panel of selected fluorescent antibodies (Table 2). The panel gave us the possibility to discard lineage-positive cell populations in the flow cytometric data, excluding T-cells (CD3+), B-cells (CD19+ and CD20+), and NK-cells (CD54+).

The gating strategy used (Fig. 1) allowed identification of several inflammatory cell populations of interest, including classical DCs type 2 (cDC2)^13,18,19^ characterized by being HLA-DR+, CD14-, CD11c high and CD1c+, classical dendritic cells type 1 (cDC1) characterized by HLA-DR+, CD14-, CD11c+, and CD141+) as well as inflammatory monocyte-derived dendritic cells (moDCs) identified by being HLA-DR+, CD14+, CD11c+, and CD1c+.

**FIG. 2.**
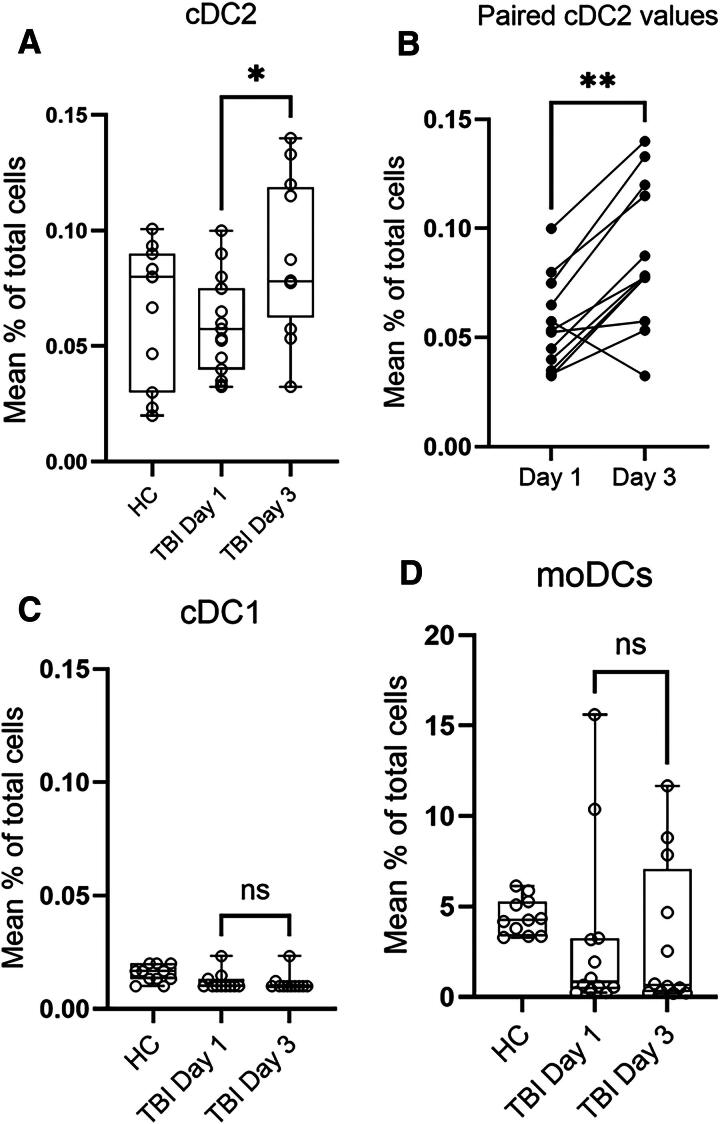
Circulating inflammatory cells in blood from healthy donors and TBI patients admitted for neurointensive care. **(A)** Levels of classical dendritic cells type 2 (cDC2s) in blood from healthy controls (HC) and TBI-patients at days 1 and 3 after arrival for neurointensive care. A significant increase in levels of cDC2 cells in TBI patients from day 1 until day 3 was found (*t*-test, *n* = 15 and 12, *p* = 0.014). **(B)** Changes over time in levels of circulating (cDC2) in traumatized patients (*n* = 12) analyzed by flow cytometry at days 1 and day 3 after admission. A significant increase (**) of cDC2s from day 1 to day 3 is evident (Wilcoxon test). **(C)** Levels of cDC1s in blood from healthy donors and TBI patients. Mann–Whitney test did not show any significant differences in TBI patients from day 1 until day 3 (*n* = 11 and *n* = 11, *p* = 0.591). **(D)** Levels of CD14+ inflammatory monocyte-derived dendritic cells (moDc) in blood from healthy donors and TBI patients. Again, no significant difference was found at day and 3 (Mann–Whitney, *n* = 12 and *n* = 12, *p* = 0.729) although a reduction compared to healthy controls may be indicated. TBI, traumatic brain injury.

In particular, cDC2 cells stand out in the dataset and showed an increase in the blood of many individual TBI patients from day 1 until day 3 after hospitalization (Fig. 2A). A test of differences in cDC2 values at day 1 and at day 3 depending on the method for preservation failed to show any statistically significant differences (*p* = 0.268 and *p* = 0.219, respectively). Analyzing the mean percentage of cDC2 cells in all patients and healthy blood donors clearly identified this population of DCs in normal blood and at similar levels 1 day after TBI but showed that circulating cDC2 cells increased significantly 3 days after admittance (Fig. 2A, *p* = 0.014). Paired levels at day 1 and day 3 in individual TBI patients (Fig. 2B) showed increases with time in 11 out of the 12 patients. For unknown reasons, only patient 6 (Table 1) had reduced levels of circulating cDC2 cells at day 3 compared to day 1 (Fig. 2B).

**FIG. 3.**
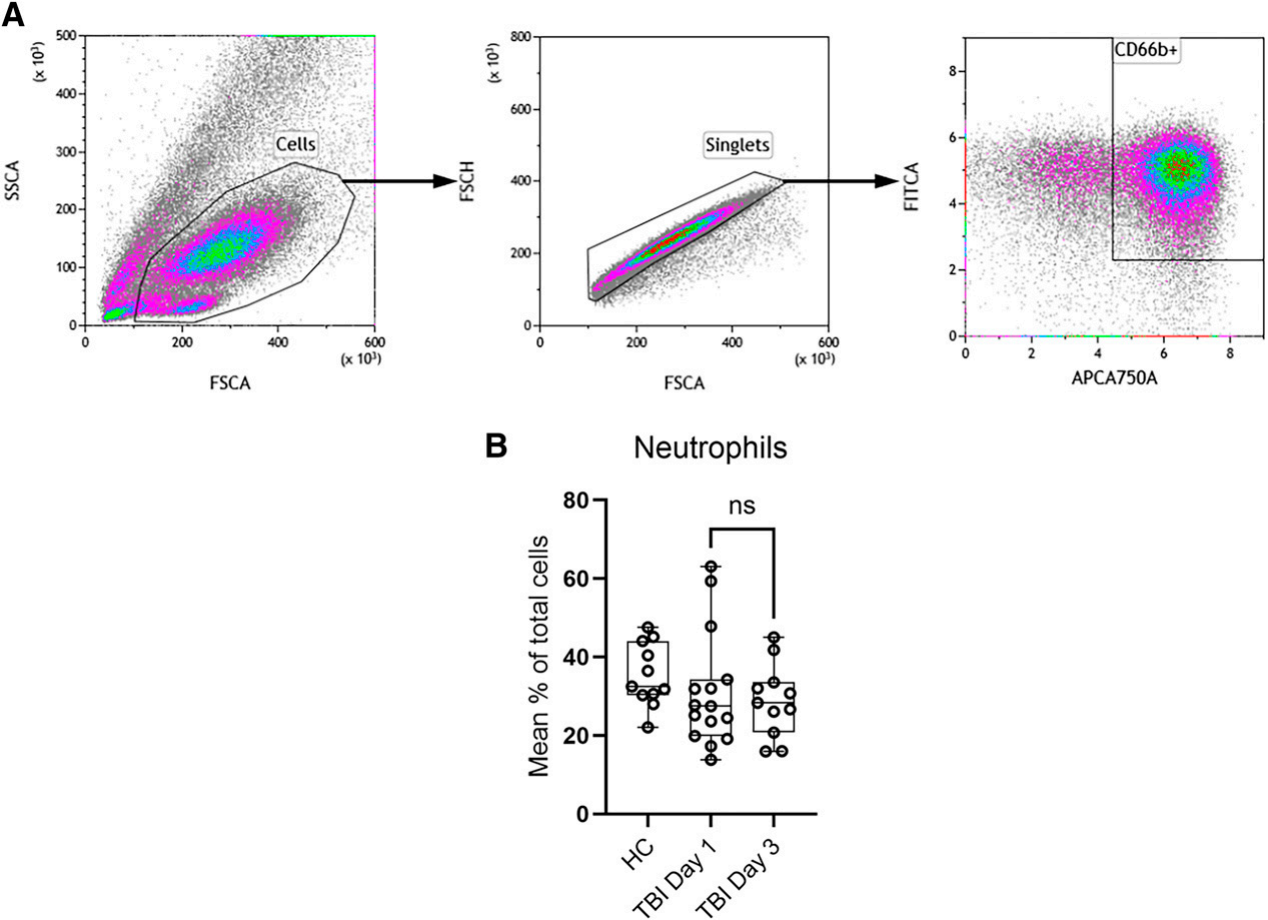
Gating strategy to detect neutrophil granulocytes (CD66b-positive cells) in peripheral blood **(A)**. Levels of neutrophils from healthy blood donors as controls (HC, *n* = 11) and TBI patients at day 1 (*n* = 15, including patient 19 sampled only at day 2) and day 3 (*n* = 11) after admission **(B).** No significant difference at day 1 and 3 was found (Mann–Whitney, *n* = 15 and *n* = 11, *p* = 0.979) although a slight reduction compared to healthy controls (HC) may be indicated at both timepoints. TBI, traumatic brain injury.

Similarly, no significant differences between Cytodelics and CryoStor CS10 were found in blood from healthy donors for cDC1, moDC, and neutrophils (*p* = 0.819, *p* = 0.183, and *p* = 0.195, respectively). Thus, further analyses lumped the two preservation methods together (Figs. 2C,D and 3B). The rare population of cDC1 cells^18–19^ did not increase in the blood of TBI patients compared to healthy controls, nor was any increase with time detected (*p* = 1.000; Fig. 2C). It is of note that the levels of circulation cDC1 cells are close to the detection limit. A similar lack of temporal changes was seen when analyzing the more abundant population of moDCs DCs (*p* = 0.540; Fig. 2D).

The high levels of neutrophilic granulocytes identified by being CD66b+ required a separate gating strategy (Fig. 3A). The average for all patients regardless of whether blood samples were collected at day 1 or day 3 did not differ significantly (*p* = 0.540) nor deviate from the levels found in random healthy volunteers (Fig. 3B). Severe TBI is often associated with an increased neutrophil count. This number may be further increased by injury to other organ systems and by infectious complications. In our cohort, the majority had an isolated TBI, and during the study period none had a significant infection. Furthermore, the timing of the sampling (days 1 and 3 after submission) may have missed a possible peak in the number of circulating neutrophils.

A slight reduction of both moDCs and neutrophiles in the TBI patients compared to the levels in healthy controls is indicated, although not statistically significant. It may reflect that these cells have extravasated from blood into tissue as a response to chemokine release due to the trauma and that the situation in blood circulation is not yet normalized at day three after the injury.

## Discussion

This study represents a phenotypic atlas of the acute response of circulating innate immune cells in patients with TBI.^6,19^ The patients were admitted to a neurointensive care unit, and the majority of blood samples were taken on day 1 and day 3 after submission. To stabilize the blood cells for flow-cytometric analyses, patients were subjected to two alternative freezing regimens—either preservation with Cytodelics or CryoStor CS10. For comparative analyses, we also collected blood from healthy blood donors. In this initial study, study, ages, sex, or background health conditions were not balanced, but we consider the results appropriate for preliminary conclusions and for considerations of future strategies.

The release of chemokines attracting inflammatory cells from the circulation to enter the brain after TBI has been demonstrated in a number of studies in human patients as well as rodents.^4,5,8,12,20–23^ In a focal TBI model in mice, we previously showed that the chemokine Ccl2 mRNA is rapidly upregulated.^9–10^ This is also the case in human TBI patients^8^ with post-traumatic cerebral contusion requiring a surgical evacuation were studied. The median interval from trauma to biopsy procedure was 44 h (range 3–360 h). Total RNA was isolated from these samples, and a ribonuclease protection assay was performed to measure the mRNA levels of several chemokines, including CCL2, a monocyte chemoattractant produced by activated astrocytes and found to be the highest expressed chemokine after TBI.^8^

In mice, the Ccl2 chemokine binds to the Ccr2 receptor (human designation CCL2 and CCR2, respectively), resulting in an invasion of DCs and other inflammatory monocytic cells to the brain parenchyma within the first few days after trauma. TBI in Ccr2-/- mice in a controlled cortical impact mode resulted in a 90% loss of monocytes invading the brain early post-TBI, compared with wild-type mice. Lack of Ccr2 improved locomotor activity and resulted in improved spatial learning and memory, significantly increased neuronal density in the CA1-CA3 regions of the hippocampus after TBI compared with wild-type mice 8 weeks after TBI.^21^ Although there was no difference in the volume of tissue loss, the data show that Ccr2 directs the majority of monocytes/DCs homing to the brain early after TBI, resulting in harmful responses.^21^ These recruited cells are expressing the integrin heterodimer Itgax/Itgb2 (CD11c/CD18), mediating binding to cell adhesion molecules (like ICAM-1) fostering transendothelial migration.^13–14^ In a rat model, peaking levels of Ccl2 and Ccr2 mRNA levels were observed in the cerebral cortex.^4^ As in mice, blocking Ccl2 activity by the Ccr2 antagonist RS504393 significantly reduced apoptotic cell death at 1–3 days post-injury and reduced the impaired cognitive performance in the Morris water maze, a test of visuospatial memory, at 3 days post-injury.

Rapid release of CCL2 to cerebrospinal fluid and serum has been shown in TBI patients, suggesting that recruitment of peripheral immune cells to the injured area is occurring also in humans.^12^ CCL2 protein levels in cerebrospinal fluid of patients with severe TBI (Glasgow Coma Scale score <8) had increased to 19 ng/mL upon admission from normal levels of 0.7 ng/mL, gradually diminishing but still significantly elevated to 3 ng/mL days 3 until 9 after admission. A mouse model of closed head injury also showed 40-fold increased levels of Ccl2 protein levels in the cerebral cortex during day one after injury, peaking at 4 and 12 h post-injury.^12^ Interestingly, in Ccl2-/- mice the same injury was manifest by delayed lesion volume, reduced astrocytosis, and lowered accumulation of inflammatory cells in the cortex but not until several weeks after the injury, again adding to the concept of long-term deleterious effects of CCL2 in TBI.

TBI patients in neurointensive care with high levels of CCL2 in serum had a significantly increased mortality. However, increased levels of CCL2 in serum were not coupled to enlarging contusions. In patients with severe diffuse TBI analyzed for CCL2 levels by cerebral microdialysis and arterial and jugular bulb plasma sampling over a 5-day period, brain extracellular fluid levels of CCL2 were significantly 11-fold higher compared to plasma levels, strongly indicating local cerebral chemokine production as part of the inflammatory response in the traumatically injured brain.^5^ Chemokines may promote neuroinflammation following TBI, thereby exacerbating secondary injury. Another study investigating the contribution of chemokines to TBI severity and clinical outcome was based on peripheral blood from 92 TBI patients on admission, and 40 recruited controls.^20^ Serum concentrations of CCL2 and other chemokines were measured by ELISA on admission. Pre-operative clinical severity was evaluated using the GCS, and clinical outcome at 90 days post-TBI was evaluated using the Glasgow Outcome Scale (GOSE). Serum concentrations of CCL2 were elevated significantly after TBI and negatively correlated with GCS and GOS scores. CCL2 was considered as an independent predictor of severity and outcome.^20^

How the injury increase of CCL2 in CSF and serum affects the levels of various populations of DCs and monocytes circulating in the blood remains less well studied. Open questions are whether brain injury lowers or depletes the limited pool of circulating DCs or if TBI stimulates increased release of inflammatory cells from the spleen, bone marrow, or lymph nodes. Here, samples taken from TBI patients were preserved in Cytodelics cryopreservation of cells, or freezing with the reagent CryoStor CS10 to stabilize blood cells for analyses by flow cytometry. DC subsets are more diversified in humans than in mice.^19^ The present study was focused on cDC2, cDC1, and moDC whereas plasmacytoid DCs and DCs type 3 were left out.^19^ All these subsets of cells were CD11c+ indicating expression of integrin alpha-X and positive for HLA-DR, suggestive of various degrees of capacity to present antigens on major histocompatibilty complex II.

Our current preliminary report is not without limitations. During the third day after injury, patients with severe TBI had a significant increase of cDC2 cells in peripheral blood, irrespective of the two different freezing methods. To assess the dynamics of the cell response with greater precision, repeated measurements over time and with larger sample sizes of the included patients would be preferable. Although the included patients were suffering from severe TBI from various causes of trauma, we cannot exclude confounding effects from peripheral injuries or prior health conditions. In addition, we have not performed any subclassification of the TBI types. An optimal study design would adjust for these factors and investigate if changes in specific innate immune cell responses predict clinical outcome.

## Conclusion

Regardless of the two different blood preservation techniques applied, antigen-presenting cDC2 cells were the only inflammatory cell population in peripheral blood that significantly increased from day 1 to day 3 in small independent cohorts of patients with moderate-severe TBI.

## Abbreviations Used

aSDH: acute subdural hematoma
BSA: bovine serum albumin
CCL: chemokine C-C motif ligand
CCR: C-C chemokine receptor
CCR2: C chemokine type 2 receptor
CD: cluster of differentiation
cDC2: classical dendritic cell type 2
cDC1: classical dendritic cell type 1
CNS: central nervous system
CT: computed tomography
DC: dendritic cell
EDH: epidural hematoma
F: female patient
FMO: fluorescent minus one
GCS: Glasgow Coma Scale
GOSE: Glasgow Outcome Scale
IVH: intraventricular hemorrhage
M: male patient
moDC: inflammatory monocyte-derived dendritic cell
NK: natural killer
NR: not reported
PBS: phosphate buffered saline
RT: room temperature
SS: single stain
TBI: traumatic brain injury
tICH: traumatic intracerebral hemorrhage
tSAH: traumatic subdural hematoma
US: unstained samples
